# SGLT2 Inhibitors—A Medical Revelation: Molecular Signaling of Canagliflozin Underlying Hypertension and Vascular Remodeling

**DOI:** 10.1161/JAHA.122.026774

**Published:** 2022-07-29

**Authors:** Takashi Yagi, Gopi K. Kolluru

**Affiliations:** ^1^ Department of Pathology LSU Health Shreveport Shreveport LA

**Keywords:** Editorials, heart failure, high blood pressure, hypertension, mechanisms, myocardial infarction, Hypertension, High Blood Pressure, Heart Failure, Myocardial Infarction, Mechanisms

Cardiovascular disease (CVD) is a major contributor to morbidity and mortality causing global economic burden to the health care system. Chronic kidney disease (CKD) is an important risk factor for the progression of CVD. CVD and CKD are interrelated diseases with reciprocated dysfunction that can ultimately lead to organ failure.^1^ Risk factors, including age, hypertension, diabetes, dyslipidemia, obesity, and family history, are mutual and can accelerate both CVD and CKD. Hypertension is a major cause of atherosclerosis that leads to severity and progression of CVD complications, including myocardial infarction, heart attack, heart failure, stroke, CKD, and eventually death.^1^ Patients with CKD have higher urinary sodium excretion, which is associated with an increased risk of CVD. An imbalance in sodium and fluid homeostasis can lead to the development of hypertension.^2^ Evidence from clinical trials show beneficial effects of antihypertensive treatments preventing future CVD events^3^; however, the underlying signaling in relation to CVD/CKD risk and mortality have been poorly understood.

Hypertension can be classified as salt‐sensitive and non–salt‐sensitive hypertension, with an estimated 50% of patients being salt sensitive. Sodium‐glucose cotransporter‐2 inhibitors (SGLT2i) are a unique class of drugs that improve glucose metabolism, decrease blood pressure, and have cardiorenal benefits in patients with diabetes.^4^ Preclinical studies on SGLT2i suggest early‐stage antihypertensive effects via natriuresis and osmotic diuresis in nondiabetic salt‐sensitive hypertensive models.^5^, ^6^, ^7^ Notably, recent findings extend their benefits not only in maintaining glucose homeostasis but also to reduce cardiovascular mortality.^8^ Protective effects of SGLT2i include reduced hypertension and better cardiac output in patients with heart failure regardless of diabetes.^5^, ^8^ However, the mechanisms underlying SGLT2i's beneficial effects remain undetermined.

Canonical transient receptor potential channels (TRPCs) are distributed in all major tissues and regulate Ca^2+^ influx, vasoconstriction, and blood pressure, and play significant roles in the cardiovascular system, neurons, pancreatic β‐cells, bone, and the immune system.^7^ Although increased endothelial cell TRPC6 expression is a hallmark of atherosclerosis and atheroprone flow shear stress,^9^ smooth muscle cell TRPC3 mediates pathological implications in the blood vessels.^7^ Additionally, TRPC3 expression was observed in peripheral blood mononuclear cells in patients with hypertension but not in normotensive subjects.^10^ Despite this evidence, the underlying mechanisms of SGLT2i lowering hypertension contributing to CVD risk^11^ and their short‐ to long‐term effects are not well understood. The current issue of the *Journal of the American Heart Association* (*JAHA*) includes a study by Zhao et al that demonstrates the mechanisms underlying how SGLT2i canagliflozin improves hypertension in the Dahl salt‐sensitive rat hypertensive model.^12^ This study reveals TRPC3 as a salt‐sensitive channel that interacts with NCX1 (sodium/calcium exchanger 1) to modulate dysfunction of vascular calcium handling and hypertension, which is significantly improved by canagliflozin treatment. Although overexpression of TRPC3 mimicked the antihypertensive effects of canagliflozin, the TRPC3 knockout nullified these effects. Mechanistically, high salt‐induced cytoplasmic calcium level and vasoconstriction, which required TRPC3, and this process could be blocked by canagliflozin.^12^ They also revealed the novel mechanism of how canagliflozin improves endothelial function in vivo and in vitro. SGLT2i TRPC3 interaction would be a new therapeutic target, but direct mechanisms between SGLT2i and TRPC3 remain unknown.

In the past decade, the awareness and number of treatment alternatives for patients with hypertension have increased in developed countries; however, ≈50% of known patients with hypertension have uncontrolled blood pressure in the United States. Similar findings have been observed in other countries, even in patients who are already under medication.^13^ This could be attributable to the failure of health care providers to initiate or intensify therapy when needed, which is defined as *clinical inertia*.^14^ There are several reasons for clinical inertia, including patient behavior and physician behavior, or both. As is well known, hypertension, diabetes, and dyslipidemia likely have clinical inertia, and SGLT2i can be a step forward as a medication to combat it. SGLT2i, including canagliflozin, dapagliflozin, and empagliflozin, have been used as antidiabetic drugs reducing glucose reabsorption in the renal proximal tubule with resultant glucosuria and lowering glucose levels in an insulin‐independent manner.^13^ SGLT2i have benefits not only in improving hyperglycemia but also in reducing hyperinsulinemia, body weight, and insulin resistance.^13^ Because SGLT2i show pleiotropic effect, SGLT2i are receiving attention as drugs for reducing cardiovascular events and improving renal function.

Increasing evidence from multiple clinical trials suggests a strong association between SGLT2i and their protection in patients with heart failure.^15^, ^16^, ^17^, ^18^, ^19^ EMPA‐REG (Empagliflozin Cardiovascular Outcome Event Trial in Type 2 Diabetes Mellitus Patients) is the first randomized, double‐blind, placebo‐controlled trial that showed that SGLT2i significantly decreased death from cardiovascular causes, nonfatal myocardial infarction, or stroke in patients with type 2 diabetes.^15^ DAPA‐HF (Dapagliflozin and Prevention of Adverse Outcomes in Heart Failure)^16^ and EMPEROR‐Reduced (Empagliflozin outcome trial in Patients With chronic heart Failure With Reduced Ejection Fraction) trials^18^ showed that SGLT2i reduced the combined risk of cardiovascular death or hospitalization in patients with heart failure with reduced ejection fraction independent of diabetes. The CANVAS (Canagliflozin cardiovascular Assessment Study) program showed that canagliflozin significantly lowered the risk of cardiovascular events in patients with type 2 diabetes and an elevated risk of CVD.^17^ Similarly, the DECLARE‐TIMI 58 (Dapagliflozin Effect on Cardiovascular Events–Thrombolysis in Myocardial Infarction 58) trial, a multicenter, randomized, double‐blind, placebo‐controlled trial, used SGLT2i dapagliflozin in patients with type 2 diabetes.^19^ These studies, along with the current study, suggest the protective effects of SGLT2i are beyond blood glucose regulation and enhanced diuretic effects.^12^ Effects of SGLT2i may involve changes including myocardial and renal metabolism, and endothelial function. Importantly, these trials confirm the SGLT2i benefits on reduced hospitalized heart failure, cardiovascular death, and all‐cause mortality in patients with heart failure with reduced ejection fraction, and renal composite outcomes.^9^, ^17^, ^19^, ^20^

Overall, Zhao et al^12^ demonstrate SGLT2i canagliflozin as an effective therapeutic measure to antagonize salt‐induced hypertension by alleviating vasoconstriction. This study identifies the mechanisms underlying the antihypertensive effects of canagliflozin via TRPC3 and NCX1 signaling beyond its canonical effect on renal natriuresis. Their study further establishes that elevated TRPC3/NCX1 represents a novel target to antagonize salt‐sensitive hypertension. However, clinical studies revealing how SGLT2i‐mediated vascular remodeling contributes to beneficial cardiovascular outcomes are warranted.

## Sources of Funding

This work was supported by the Pilot grant to Dr Kolluru from Institutional Development Award from the National Institutes of General Medical Sciences of the National Institutes of Health (NIH) under grant number P20GM121307

## Disclosures

None.
